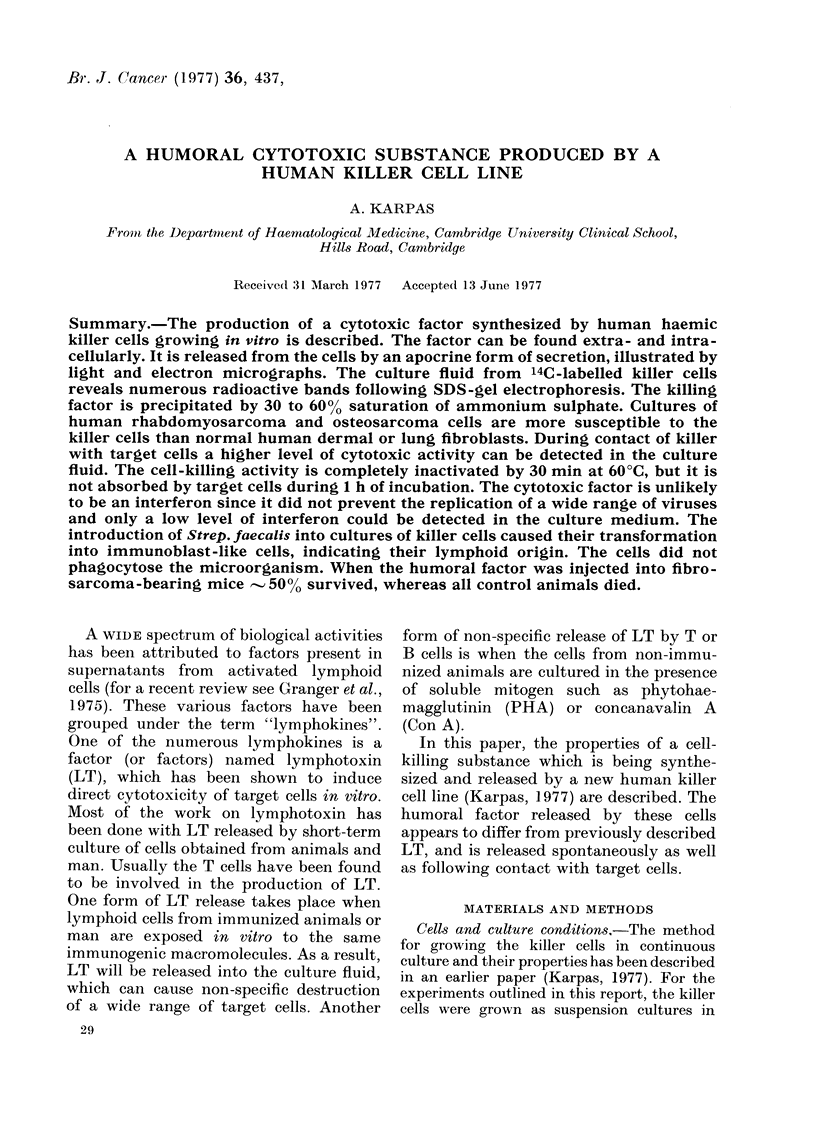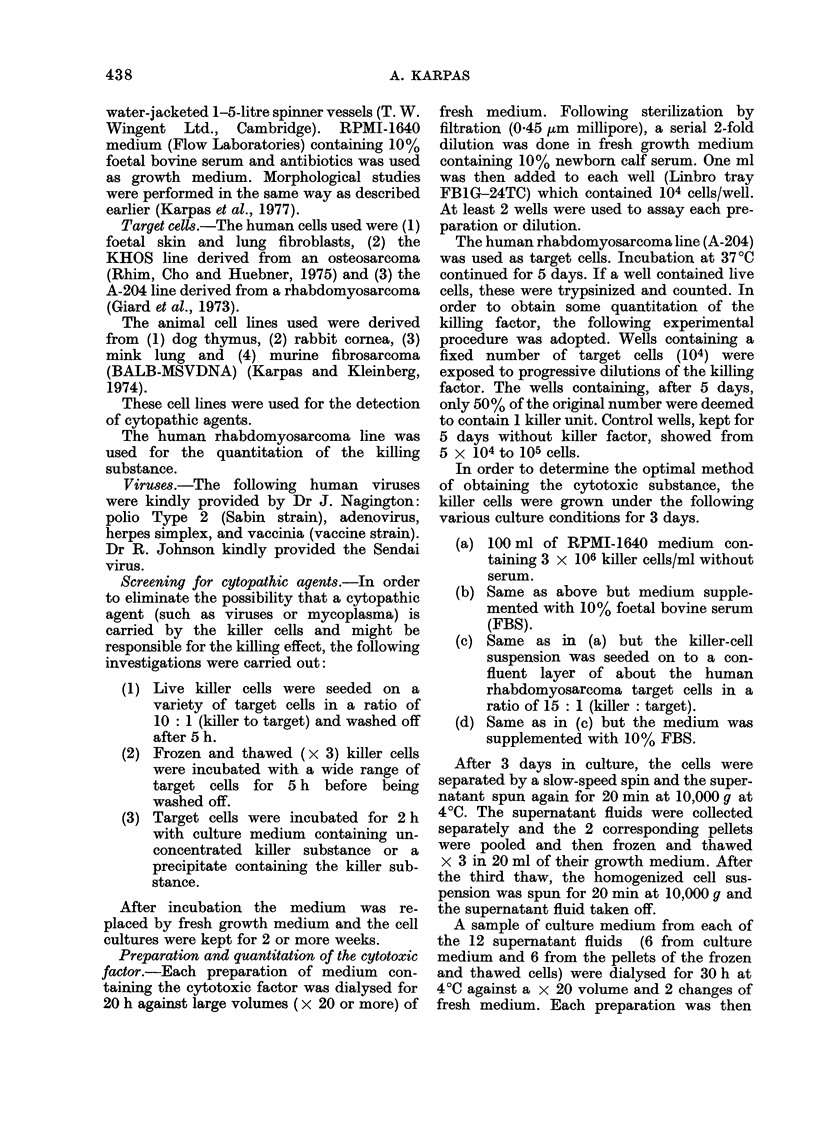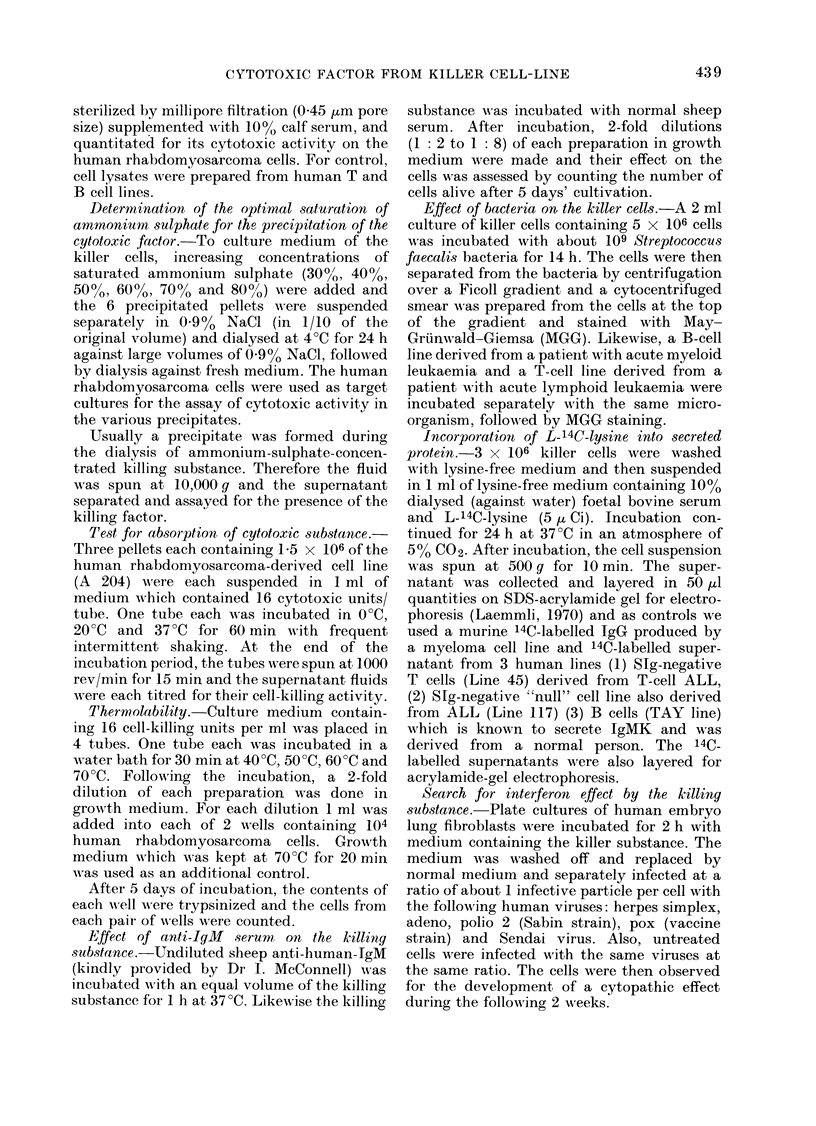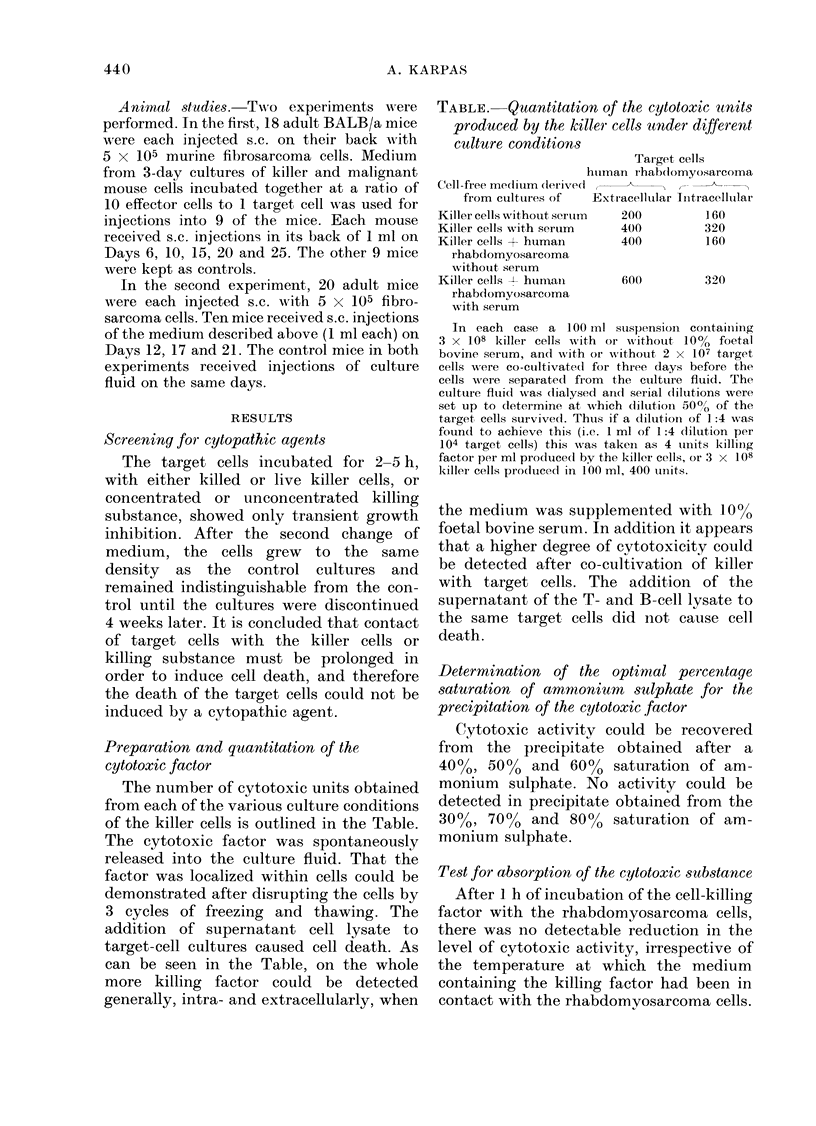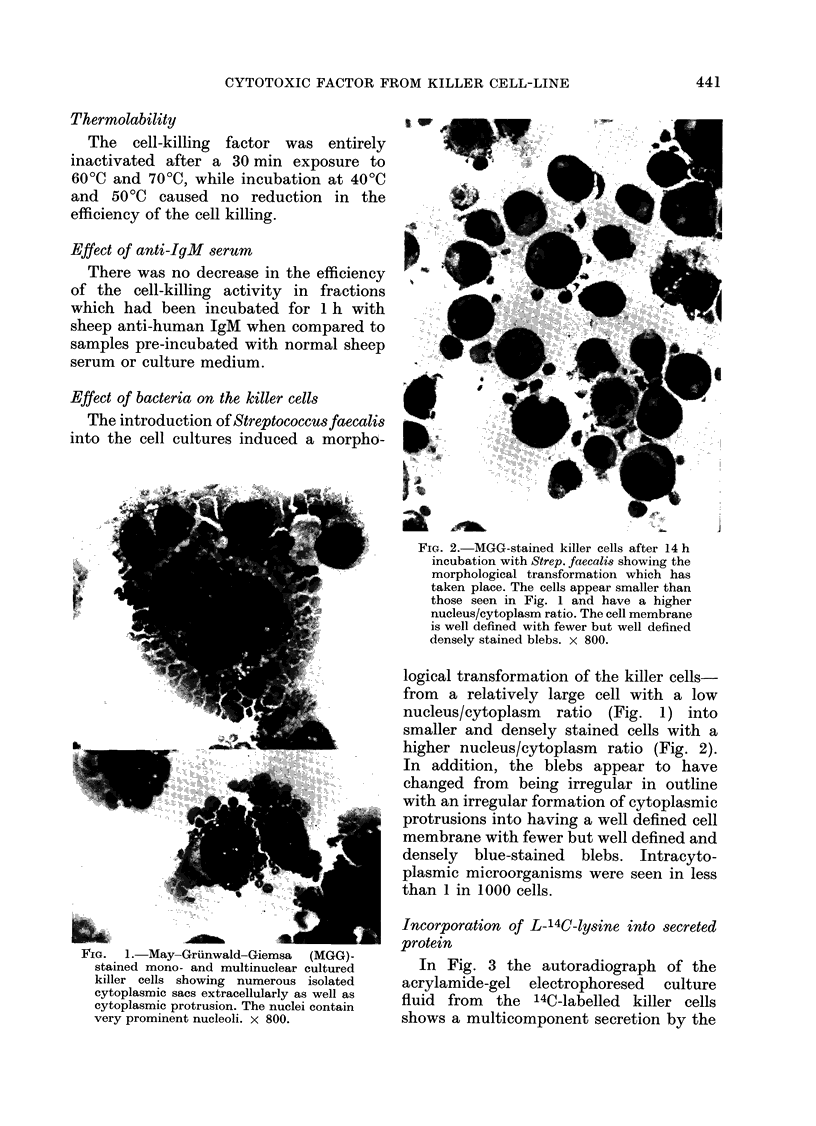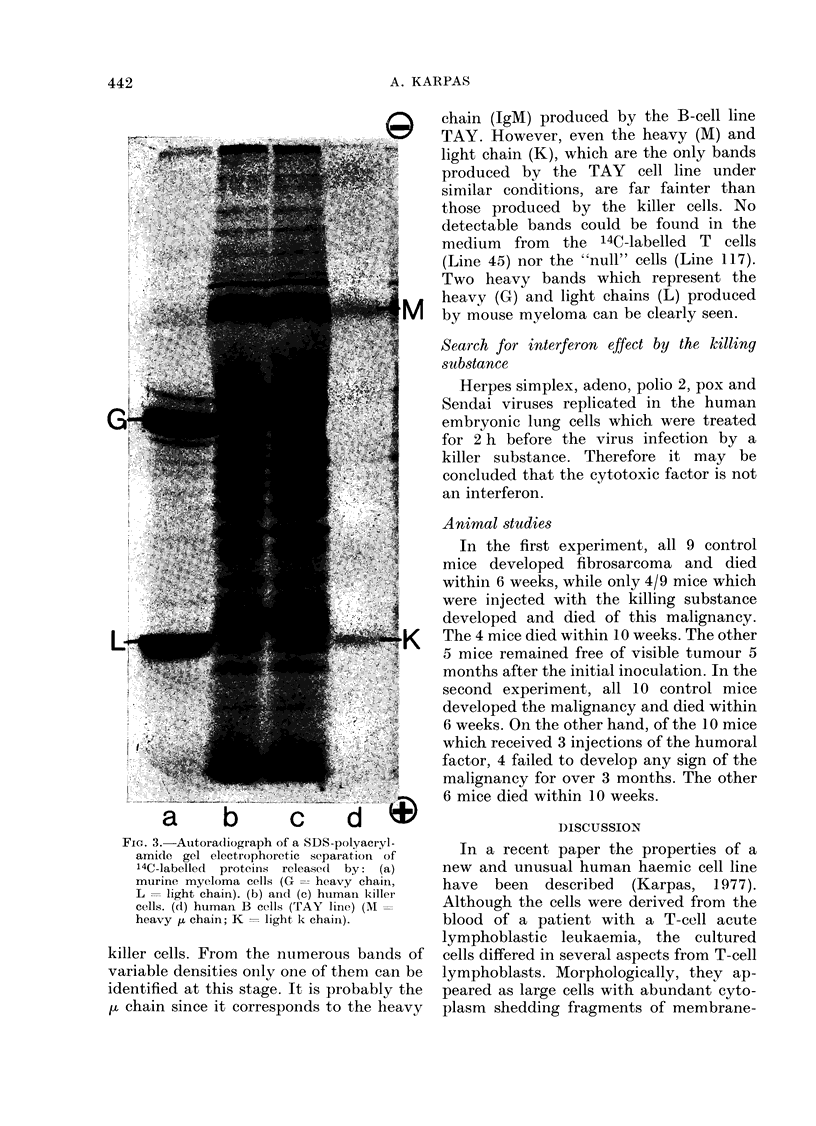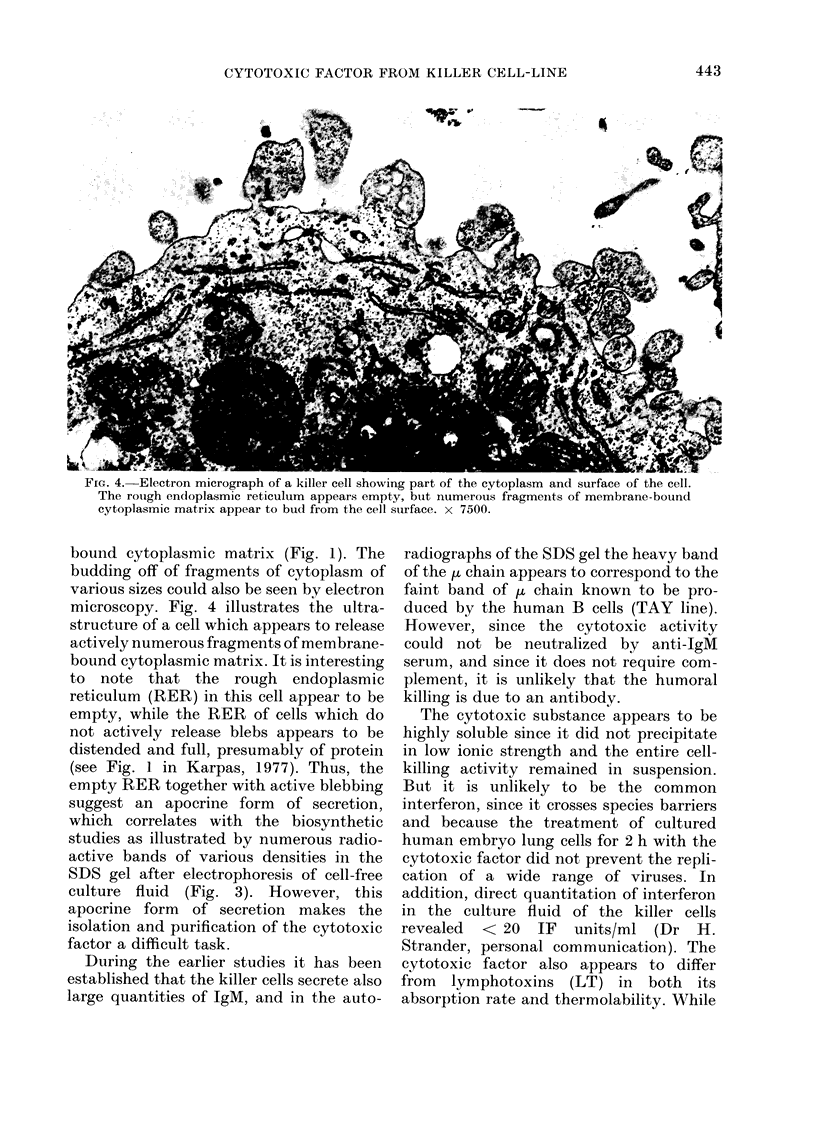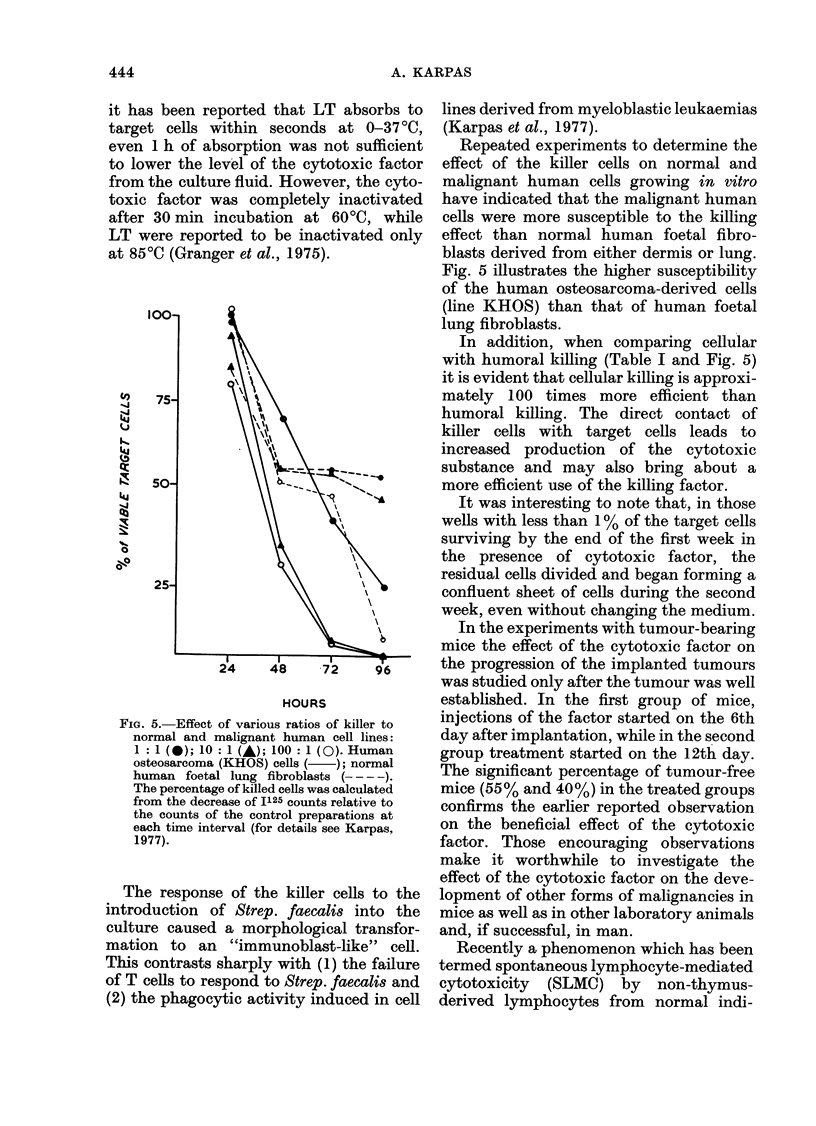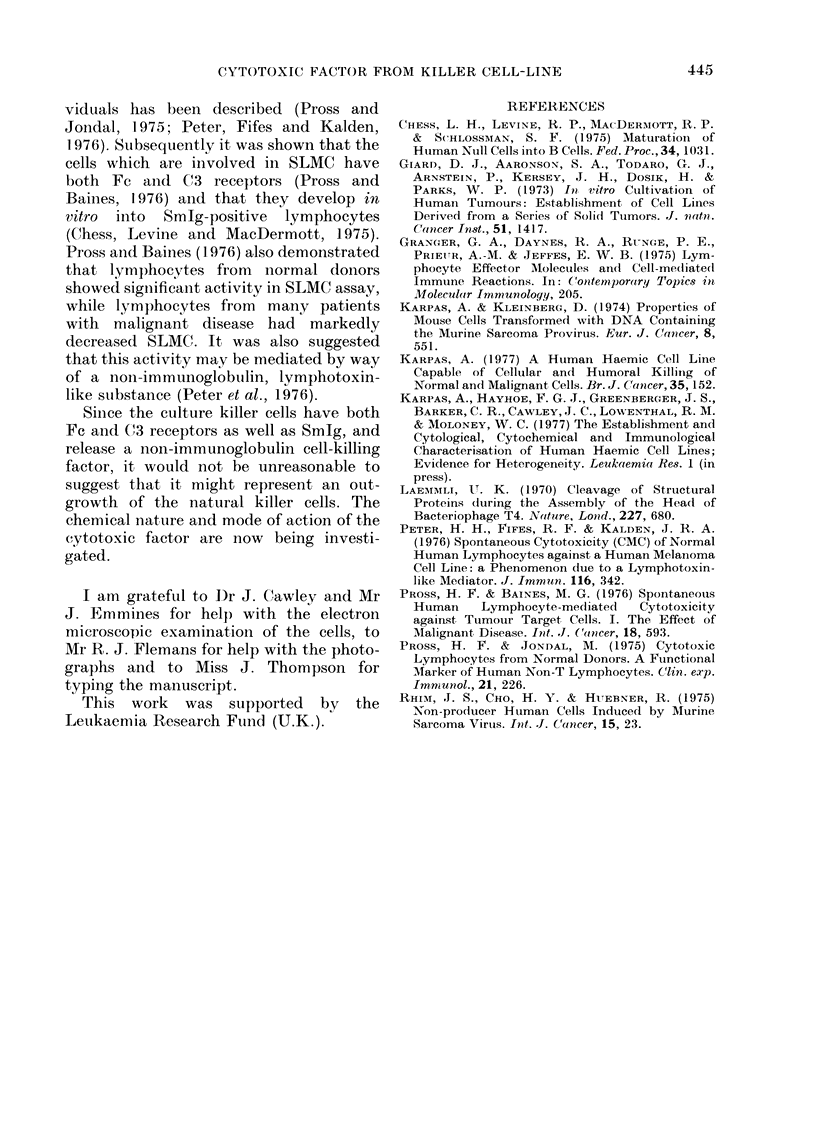# A humoral cytotoxic substance produced by a human killer cell line.

**DOI:** 10.1038/bjc.1977.212

**Published:** 1977-10

**Authors:** A. Karpas

## Abstract

**Images:**


*Br. J. Cancer* (1977) **36**, 437,

### A HUMORAL CYTOTOXIC SUBSTANCE PRODUCED BY A HUMAN KILLER CELL LINE

A. KARPAS

*From the Department of Haematological Medicine, Cambridge University Clinical School, Hills Road, Cambridge*

Received 31 March 1977 Accepted 13 June 1977

**Summary.—The production of a cytotoxic factor synthesized by human haemic killer cells growing *in vitro* is described. The factor can be found extra- and intracellularly. It is released from the cells by an apocrine form of secretion, illustrated by light and electron micrographs. The culture fluid from $^{14}C$-labelled killer cells reveals numerous radioactive bands following SDS-gel electrophoresis. The killing factor is precipitated by 30 to 60% saturation of ammonium sulphate. Cultures of human rhabdomyosarcoma and osteosarcoma cells are more susceptible to the killer cells than normal human dermal or lung fibroblasts. During contact of killer with target cells a higher level of cytotoxic activity can be detected in the culture fluid. The cell-killing activity is completely inactivated by 30 min at 60°C, but it is not absorbed by target cells during 1 h of incubation. The cytotoxic factor is unlikely to be an interferon since it did not prevent the replication of a wide range of viruses and only a low level of interferon could be detected in the culture medium. The introduction of *Strep. faecalis* into cultures of killer cells caused their transformation into immunoblast-like cells, indicating their lymphoid origin. The cells did not phagocytose the microorganism. When the humoral factor was injected into fibrosarcoma-bearing mice ~50% survived, whereas all control animals died.**

A WIDE spectrum of biological activities has been attributed to factors present in supernatants from activated lymphoid cells (for a recent review see Granger *et al.*, 1975). These various factors have been grouped under the term "lymphokines". One of the numerous lymphokines is a factor (or factors) named lymphotoxin (LT), which has been shown to induce direct cytotoxicity of target cells *in vitro.* Most of the work on lymphotoxin has been done with LT released by short-term culture of cells obtained from animals and man. Usually the T cells have been found to be involved in the production of LT. One form of LT release takes place when lymphoid cells from immunized animals or man are exposed *in vitro* to the same immunogenic macromolecules. As a result, LT will be released into the culture fluid, which can cause non-specific destruction of a wide range of target cells. Another form of non-specific release of LT by T or B cells is when the cells from non-immunized animals are cultured in the presence of soluble mitogen such as phytohaemagglutinin (PHA) or concanavalin A (Con A).

In this paper, the properties of a cell-killing substance which is being synthesized and released by a new human killer cell line (Karpas, 1977) are described. The humoral factor released by these cells appears to differ from previously described LT, and is released spontaneously as well as following contact with target cells.

### MATERIALS AND METHODS

*Cells and culture conditions.*—The method for growing the killer cells in continuous culture and their properties has been described in an earlier paper (Karpas, 1977). For the experiments outlined in this report, the killer cells were grown as suspension cultures in water-jacketed 1–5-litre spinner vessels (T. W. Wingent Ltd., Cambridge). RPMI-1640 medium (Flow Laboratories) containing 10% foetal bovine serum and antibiotics was used as growth medium. Morphological studies were performed in the same way as described earlier (Karpas *et al.*, 1977).

*Target cells.*—The human cells used were (1) foetal skin and lung fibroblasts, (2) the KHOS line derived from an osteosarcoma (Rhim, Cho and Huebner, 1975) and (3) the A-204 line derived from a rhabdomyosarcoma (Giard *et al.*, 1973).

The animal cell lines used were derived from (1) dog thymus, (2) rabbit cornea, (3) mink lung and (4) murine fibrosarcoma (BALB-MSVDNA) (Karpas and Kleinberg, 1974).

These cell lines were used for the detection of cytopathic agents.

The human rhabdomyosarcoma line was used for the quantitation of the killing substance.

*Viruses.*—The following human viruses were kindly provided by Dr J. Nagington: polio Type 2 (Sabin strain), adenovirus, herpes simplex, and vaccinia (vaccine strain). Dr R. Johnson kindly provided the Sendai virus.

*Screening for cytopathic agents.*—In order to eliminate the possibility that a cytopathic agent (such as viruses or mycoplasma) is carried by the killer cells and might be responsible for the killing effect, the following investigations were carried out:

(1) Live killer cells were seeded on a variety of target cells in a ratio of 10 : 1 (killer to target) and washed off after 5 h.
(2) Frozen and thawed ($\times$ 3) killer cells were incubated with a wide range of target cells for 5 h before being washed off.
(3) Target cells were incubated for 2 h with culture medium containing unconcentrated killer substance or a precipitate containing the killer substance.

After incubation the medium was replaced by fresh growth medium and the cell cultures were kept for 2 or more weeks.

*Preparation and quantitation of the cytotoxic factor.*—Each preparation of medium containing the cytotoxic factor was dialysed for 20 h against large volumes ($\times$ 20 or more) of fresh medium. Following sterilization by filtration (0·45 $\mu$m millipore), a serial 2-fold dilution was done in fresh growth medium containing 10% newborn calf serum. One ml was then added to each well (Linbro tray FB1G–24TC) which contained $10^4$ cells/well. At least 2 wells were used to assay each preparation or dilution.

The human rhabdomyosarcoma line (A-204) was used as target cells. Incubation at 37 °C continued for 5 days. If a well contained live cells, these were trypsinized and counted. In order to obtain some quantitation of the killing factor, the following experimental procedure was adopted. Wells containing a fixed number of target cells ($10^4$) were exposed to progressive dilutions of the killing factor. The wells containing, after 5 days, only 50% of the original number were deemed to contain 1 killer unit. Control wells, kept for 5 days without killer factor, showed from $5 \times 10^4$ to $10^5$ cells.

In order to determine the optimal method of obtaining the cytotoxic substance, the killer cells were grown under the following various culture conditions for 3 days.

(a) 100 ml of RPMI-1640 medium containing $3 \times 10^6$ killer cells/ml without serum.
(b) Same as above but medium supplemented with 10% foetal bovine serum (FBS).
(c) Same as in (a) but the killer-cell suspension was seeded on to a confluent layer of about the human rhabdomyosarcoma target cells in a ratio of 15 : 1 (killer : target).
(d) Same as in (c) but the medium was supplemented with 10% FBS.

After 3 days in culture, the cells were separated by a slow-speed spin and the supernatant spun again for 20 min at 10,000 $g$ at 4°C. The supernatant fluids were collected separately and the 2 corresponding pellets were pooled and then frozen and thawed $\times$ 3 in 20 ml of their growth medium. After the third thaw, the homogenized cell suspension was spun for 20 min at 10,000 $g$ and the supernatant fluid taken off.

A sample of culture medium from each of the 12 supernatant fluids (6 from culture medium and 6 from the pellets of the frozen and thawed cells) were dialysed for 30 h at 4 °C against a $\times$ 20 volume and 2 changes of fresh medium. Each preparation was then sterilized by millipore filtration (0·45 $\mu$m pore size) supplemented with 10% calf serum, and quantitated for its cytotoxic activity on the human rhabdomyosarcoma cells. For control, cell lysates were prepared from human T and B cell lines.

*Determination of the optimal saturation of ammonium sulphate for the precipitation of the cytotoxic factor.*—To culture medium of the killer cells, increasing concentrations of saturated ammonium sulphate (30%, 40%, 50%, 60%, 70% and 80%) were added and the 6 precipitated pellets were suspended separately in 0·9% NaCl (in 1/10 of the original volume) and dialysed at 4°C for 24 h against large volumes of 0·9% NaCl, followed by dialysis against fresh medium. The human rhabdomyosarcoma cells were used as target cultures for the assay of cytotoxic activity in the various precipitates.

Usually a precipitate was formed during the dialysis of ammonium-sulphate-concentrated killing substance. Therefore the fluid was spun at 10,000 *g* and the supernatant separated and assayed for the presence of the killing factor.

*Test for absorption of cytotoxic substance.*—Three pellets each containing $1{\cdot}5 \times 10^6$ of the human rhabdomyosarcoma-derived cell line (A 204) were each suspended in 1 ml of medium which contained 16 cytotoxic units/tube. One tube each was incubated in 0°C, 20°C and 37°C for 60 min with frequent intermittent shaking. At the end of the incubation period, the tubes were spun at 1000 rev/min for 15 min and the supernatant fluids were each titred for their cell-killing activity.

*Thermolability.*—Culture medium containing 16 cell-killing units per ml was placed in 4 tubes. One tube each was incubated in a water bath for 30 min at 40°C, 50°C, 60°C and 70°C. Following the incubation, a 2-fold dilution of each preparation was done in growth medium. For each dilution 1 ml was added into each of 2 wells containing $10^4$ human rhabdomyosarcoma cells. Growth medium which was kept at 70°C for 20 min was used as an additional control.

After 5 days of incubation, the contents of each well were trypsinized and the cells from each pair of wells were counted.

*Effect of anti-IgM serum on the killing substance.*—Undiluted sheep anti-human-IgM (kindly provided by Dr I. McConnell) was incubated with an equal volume of the killing substance for 1 h at 37°C. Likewise the killing substance was incubated with normal sheep serum. After incubation, 2-fold dilutions (1 : 2 to 1 : 8) of each preparation in growth medium were made and their effect on the cells was assessed by counting the number of cells alive after 5 days' cultivation.

*Effect of bacteria on the killer cells.*—A 2 ml culture of killer cells containing $5 \times 10^6$ cells was incubated with about $10^9$ *Streptococcus faecalis* bacteria for 14 h. The cells were then separated from the bacteria by centrifugation over a Ficoll gradient and a cytocentrifuged smear was prepared from the cells at the top of the gradient and stained with May–Grünwald–Giemsa (MGG). Likewise, a B-cell line derived from a patient with acute myeloid leukaemia and a T-cell line derived from a patient with acute lymphoid leukaemia were incubated separately with the same microorganism, followed by MGG staining.

*Incorporation of L-$^{14}$C-lysine into secreted protein.*—$3 \times 10^6$ killer cells were washed with lysine-free medium and then suspended in 1 ml of lysine-free medium containing 10% dialysed (against water) foetal bovine serum and L-$^{14}$C-lysine (5 $\mu$ Ci). Incubation continued for 24 h at 37°C in an atmosphere of 5% $CO_2$. After incubation, the cell suspension was spun at 500 *g* for 10 min. The supernatant was collected and layered in 50 $\mu$l quantities on SDS-acrylamide gel for electrophoresis (Laemmli, 1970) and as controls we used a murine $^{14}$C-labelled IgG produced by a myeloma cell line and $^{14}$C-labelled supernatant from 3 human lines (1) SIg-negative T cells (Line 45) derived from T-cell ALL, (2) SIg-negative "null" cell line also derived from ALL (Line 117) (3) B cells (TAY line) which is known to secrete IgMK and was derived from a normal person. The $^{14}$C-labelled supernatants were also layered for acrylamide-gel electrophoresis.

*Search for interferon effect by the killing substance.*—Plate cultures of human embryo lung fibroblasts were incubated for 2 h with medium containing the killer substance. The medium was washed off and replaced by normal medium and separately infected at a ratio of about 1 infective particle per cell with the following human viruses: herpes simplex, adeno, polio 2 (Sabin strain), pox (vaccine strain) and Sendai virus. Also, untreated cells were infected with the same viruses at the same ratio. The cells were then observed for the development of a cytopathic effect during the following 2 weeks.

*Animal studies.*—Two experiments were performed. In the first, 18 adult BALB/a mice were each injected s.c. on their back with $5 \times 10^5$ murine fibrosarcoma cells. Medium from 3-day cultures of killer and malignant mouse cells incubated together at a ratio of 10 effector cells to 1 target cell was used for injections into 9 of the mice. Each mouse received s.c. injections in its back of 1 ml on Days 6, 10, 15, 20 and 25. The other 9 mice were kept as controls.

In the second experiment, 20 adult mice were each injected s.c. with $5 \times 10^5$ fibrosarcoma cells. Ten mice received s.c. injections of the medium described above (1 ml each) on Days 12, 17 and 21. The control mice in both experiments received injections of culture fluid on the same days.

### RESULTS

### *Screening for cytopathic agents*

The target cells incubated for 2–5 h, with either killed or live killer cells, or concentrated or unconcentrated killing substance, showed only transient growth inhibition. After the second change of medium, the cells grew to the same density as the control cultures and remained indistinguishable from the control until the cultures were discontinued 4 weeks later. It is concluded that contact of target cells with the killer cells or killing substance must be prolonged in order to induce cell death, and therefore the death of the target cells could not be induced by a cytopathic agent.

### *Preparation and quantitation of the cytotoxic factor*

The number of cytotoxic units obtained from each of the various culture conditions of the killer cells is outlined in the Table. The cytotoxic factor was spontaneously released into the culture fluid. That the factor was localized within cells could be demonstrated after disrupting the cells by 3 cycles of freezing and thawing. The addition of supernatant cell lysate to target-cell cultures caused cell death. As can be seen in the Table, on the whole more killing factor could be detected generally, intra- and extracellularly, when

TABLE.—*Quantitation of the cytotoxic units produced by the killer cells under different culture conditions*

| Cell-free medium derived from cultures of | Target cells human rhabdomyosarcoma: Extracellular | Intracellular |
|---|---|---|
| Killer cells without serum | 200 | 160 |
| Killer cells with serum | 400 | 320 |
| Killer cells + human rhabdomyosarcoma without serum | 400 | 160 |
| Killer cells + human rhabdomyosarcoma with serum | 600 | 320 |

In each case a 100 ml suspension containing $3 \times 10^8$ killer cells with or without 10% foetal bovine serum, and with or without $2 \times 10^7$ target cells were co-cultivated for three days before the cells were separated from the culture fluid. The culture fluid was dialysed and serial dilutions were set up to determine at which dilution 50% of the target cells survived. Thus if a dilution of 1:4 was found to achieve this (i.e. 1 ml of 1:4 dilution per $10^4$ target cells) this was taken as 4 units killing factor per ml produced by the killer cells, or $3 \times 10^8$ killer cells produced in 100 ml, 400 units.

the medium was supplemented with 10% foetal bovine serum. In addition it appears that a higher degree of cytotoxicity could be detected after co-cultivation of killer with target cells. The addition of the supernatant of the T- and B-cell lysate to the same target cells did not cause cell death.

### *Determination of the optimal percentage saturation of ammonium sulphate for the precipitation of the cytotoxic factor*

Cytotoxic activity could be recovered from the precipitate obtained after a 40%, 50% and 60% saturation of ammonium sulphate. No activity could be detected in precipitate obtained from the 30%, 70% and 80% saturation of ammonium sulphate.

### *Test for absorption of the cytotoxic substance*

After 1 h of incubation of the cell-killing factor with the rhabdomyosarcoma cells, there was no detectable reduction in the level of cytotoxic activity, irrespective of the temperature at which the medium containing the killing factor had been in contact with the rhabdomyosarcoma cells.

*Thermolability*

The cell-killing factor was entirely inactivated after a 30 min exposure to 60°C and 70°C, while incubation at 40°C and 50°C caused no reduction in the efficiency of the cell killing.

*Effect of anti-IgM serum*

There was no decrease in the efficiency of the cell-killing activity in fractions which had been incubated for 1 h with sheep anti-human IgM when compared to samples pre-incubated with normal sheep serum or culture medium.

*Effect of bacteria on the killer cells*

The introduction of *Streptococcus faecalis* into the cell cultures induced a morpho-

FIG. 1.—May–Grünwald–Giemsa (MGG)-stained mono- and multinuclear cultured killer cells showing numerous isolated cytoplasmic sacs extracellularly as well as cytoplasmic protrusion. The nuclei contain very prominent nucleoli. × 800.

FIG. 2.—MGG-stained killer cells after 14 h incubation with *Strep. faecalis* showing the morphological transformation which has taken place. The cells appear smaller than those seen in Fig. 1 and have a higher nucleus/cytoplasm ratio. The cell membrane is well defined with fewer but well defined densely stained blebs. × 800.

logical transformation of the killer cells—from a relatively large cell with a low nucleus/cytoplasm ratio (Fig. 1) into smaller and densely stained cells with a higher nucleus/cytoplasm ratio (Fig. 2). In addition, the blebs appear to have changed from being irregular in outline with an irregular formation of cytoplasmic protrusions into having a well defined cell membrane with fewer but well defined and densely blue-stained blebs. Intracytoplasmic microorganisms were seen in less than 1 in 1000 cells.

*Incorporation of L-$^{14}$C-lysine into secreted protein*

In Fig. 3 the autoradiograph of the acrylamide-gel electrophoresed culture fluid from the $^{14}$C-labelled killer cells shows a multicomponent secretion by the

FIG. 3.—Autoradiograph of a SDS-polyacrylamide gel electrophoretic separation of $^{14}C$-labelled proteins released by: (a) murine myeloma cells (G = heavy chain, L = light chain). (b) and (c) human killer cells. (d) human B cells (TAY line) (M = heavy $\mu$ chain; K = light k chain).

killer cells. From the numerous bands of variable densities only one of them can be identified at this stage. It is probably the $\mu$ chain since it corresponds to the heavy chain (IgM) produced by the B-cell line TAY. However, even the heavy (M) and light chain (K), which are the only bands produced by the TAY cell line under similar conditions, are far fainter than those produced by the killer cells. No detectable bands could be found in the medium from the $^{14}C$-labelled T cells (Line 45) nor the "null" cells (Line 117). Two heavy bands which represent the heavy (G) and light chains (L) produced by mouse myeloma can be clearly seen.

*Search for interferon effect by the killing substance*

Herpes simplex, adeno, polio 2, pox and Sendai viruses replicated in the human embryonic lung cells which were treated for 2 h before the virus infection by a killer substance. Therefore it may be concluded that the cytotoxic factor is not an interferon.

*Animal studies*

In the first experiment, all 9 control mice developed fibrosarcoma and died within 6 weeks, while only 4/9 mice which were injected with the killing substance developed and died of this malignancy. The 4 mice died within 10 weeks. The other 5 mice remained free of visible tumour 5 months after the initial inoculation. In the second experiment, all 10 control mice developed the malignancy and died within 6 weeks. On the other hand, of the 10 mice which received 3 injections of the humoral factor, 4 failed to develop any sign of the malignancy for over 3 months. The other 6 mice died within 10 weeks.

### DISCUSSION

In a recent paper the properties of a new and unusual human haemic cell line have been described (Karpas, 1977). Although the cells were derived from the blood of a patient with a T-cell acute lymphoblastic leukaemia, the cultured cells differed in several aspects from T-cell lymphoblasts. Morphologically, they appeared as large cells with abundant cytoplasm shedding fragments of membrane-

FIG. 4.—Electron micrograph of a killer cell showing part of the cytoplasm and surface of the cell. The rough endoplasmic reticulum appears empty, but numerous fragments of membrane-bound cytoplasmic matrix appear to bud from the cell surface. × 7500.

bound cytoplasmic matrix (Fig. 1). The budding off of fragments of cytoplasm of various sizes could also be seen by electron microscopy. Fig. 4 illustrates the ultrastructure of a cell which appears to release actively numerous fragments of membrane-bound cytoplasmic matrix. It is interesting to note that the rough endoplasmic reticulum (RER) in this cell appear to be empty, while the RER of cells which do not actively release blebs appears to be distended and full, presumably of protein (see Fig. 1 in Karpas, 1977). Thus, the empty RER together with active blebbing suggest an apocrine form of secretion, which correlates with the biosynthetic studies as illustrated by numerous radioactive bands of various densities in the SDS gel after electrophoresis of cell-free culture fluid (Fig. 3). However, this apocrine form of secretion makes the isolation and purification of the cytotoxic factor a difficult task.

During the earlier studies it has been established that the killer cells secrete also large quantities of IgM, and in the autoradiographs of the SDS gel the heavy band of the $\mu$ chain appears to correspond to the faint band of $\mu$ chain known to be produced by the human B cells (TAY line). However, since the cytotoxic activity could not be neutralized by anti-IgM serum, and since it does not require complement, it is unlikely that the humoral killing is due to an antibody.

The cytotoxic substance appears to be highly soluble since it did not precipitate in low ionic strength and the entire cell-killing activity remained in suspension. But it is unlikely to be the common interferon, since it crosses species barriers and because the treatment of cultured human embryo lung cells for 2 h with the cytotoxic factor did not prevent the replication of a wide range of viruses. In addition, direct quantitation of interferon in the culture fluid of the killer cells revealed $< 20$ IF units/ml (Dr H. Strander, personal communication). The cytotoxic factor also appears to differ from lymphotoxins (LT) in both its absorption rate and thermolability. While it has been reported that LT absorbs to target cells within seconds at 0–37°C, even 1 h of absorption was not sufficient to lower the level of the cytotoxic factor from the culture fluid. However, the cytotoxic factor was completely inactivated after 30 min incubation at 60°C, while LT were reported to be inactivated only at 85°C (Granger *et al.*, 1975).

FIG. 5.—Effect of various ratios of killer to normal and malignant human cell lines: 1 : 1 (●); 10 : 1 (▲); 100 : 1 (○). Human osteosarcoma (KHOS) cells (——); normal human foetal lung fibroblasts (– – – –). The percentage of killed cells was calculated from the decrease of $I^{125}$ counts relative to the counts of the control preparations at each time interval (for details see Karpas, 1977).

The response of the killer cells to the introduction of *Strep. faecalis* into the culture caused a morphological transformation to an "immunoblast-like" cell. This contrasts sharply with (1) the failure of T cells to respond to *Strep. faecalis* and (2) the phagocytic activity induced in cell lines derived from myeloblastic leukaemias (Karpas *et al.*, 1977).

Repeated experiments to determine the effect of the killer cells on normal and malignant human cells growing *in vitro* have indicated that the malignant human cells were more susceptible to the killing effect than normal human foetal fibroblasts derived from either dermis or lung. Fig. 5 illustrates the higher susceptibility of the human osteosarcoma-derived cells (line KHOS) than that of human foetal lung fibroblasts.

In addition, when comparing cellular with humoral killing (Table I and Fig. 5) it is evident that cellular killing is approximately 100 times more efficient than humoral killing. The direct contact of killer cells with target cells leads to increased production of the cytotoxic substance and may also bring about a more efficient use of the killing factor.

It was interesting to note that, in those wells with less than 1% of the target cells surviving by the end of the first week in the presence of cytotoxic factor, the residual cells divided and began forming a confluent sheet of cells during the second week, even without changing the medium.

In the experiments with tumour-bearing mice the effect of the cytotoxic factor on the progression of the implanted tumours was studied only after the tumour was well established. In the first group of mice, injections of the factor started on the 6th day after implantation, while in the second group treatment started on the 12th day. The significant percentage of tumour-free mice (55% and 40%) in the treated groups confirms the earlier reported observation on the beneficial effect of the cytotoxic factor. Those encouraging observations make it worthwhile to investigate the effect of the cytotoxic factor on the development of other forms of malignancies in mice as well as in other laboratory animals and, if successful, in man.

Recently a phenomenon which has been termed spontaneous lymphocyte-mediated cytotoxicity (SLMC) by non-thymus-derived lymphocytes from normal individuals has been described (Pross and Jondal, 1975; Peter, Fifes and Kalden, 1976). Subsequently it was shown that the cells which are involved in SLMC have both Fc and C3 receptors (Pross and Baines, 1976) and that they develop *in vitro* into SmIg-positive lymphocytes (Chess, Levine and MacDermott, 1975). Pross and Baines (1976) also demonstrated that lymphocytes from normal donors showed significant activity in SLMC assay, while lymphocytes from many patients with malignant disease had markedly decreased SLMC. It was also suggested that this activity may be mediated by way of a non-immunoglobulin, lymphotoxin-like substance (Peter *et al.*, 1976).

Since the culture killer cells have both Fc and C3 receptors as well as SmIg, and release a non-immunoglobulin cell-killing factor, it would not be unreasonable to suggest that it might represent an outgrowth of the natural killer cells. The chemical nature and mode of action of the cytotoxic factor are now being investigated.

I am grateful to Dr J. Cawley and Mr J. Emmines for help with the electron microscopic examination of the cells, to Mr R. J. Flemans for help with the photographs and to Miss J. Thompson for typing the manuscript.

This work was supported by the Leukaemia Research Fund (U.K.).

### REFERENCES

Chess, L. H., Levine, R. P., MacDermott, R. P. & Schlossman, S. F. (1975) Maturation of Human Null Cells into B Cells. *Fed. Proc.*, **34**, 1031.

Giard, D. J., Aaronson, S. A., Todaro, G. J., Arnstein, P., Kersey, J. H., Dosik, H. & Parks, W. P. (1973) *In vitro* Cultivation of Human Tumours: Establishment of Cell Lines Derived from a Series of Solid Tumors. *J. natn. Cancer Inst.*, **51**, 1417.

Granger, G. A., Daynes, R. A., Runge, P. E., Prieur, A.-M. & Jeffes, E. W. B. (1975) Lymphocyte Effector Molecules and Cell-mediated Immune Reactions. In: *Contemporary Topics in Molecular Immunology*, 205.

Karpas, A. & Kleinberg, D. (1974) Properties of Mouse Cells Transformed with DNA Containing the Murine Sarcoma Provirus. *Eur. J. Cancer*, **8**, 551.

Karpas, A. (1977) A Human Haemic Cell Line Capable of Cellular and Humoral Killing of Normal and Malignant Cells. *Br. J. Cancer*, **35**, 152.

Karpas, A., Hayhoe, F. G. J., Greenberger, J. S., Barker, C. R., Cawley, J. C., Lowenthal, R. M. & Moloney, W. C. (1977) The Establishment and Cytological, Cytochemical and Immunological Characterisation of Human Haemic Cell Lines; Evidence for Heterogeneity. *Leukaemia Res.* 1 (in press).

Laemmli, U. K. (1970) Cleavage of Structural Proteins during the Assembly of the Head of Bacteriophage T4. *Nature, Lond.*, **227**, 680.

Peter, H. H., Fifes, R. F. & Kalden, J. R. A. (1976) Spontaneous Cytotoxicity (CMC) of Normal Human Lymphocytes against a Human Melanoma Cell Line: a Phenomenon due to a Lymphotoxin-like Mediator. *J. Immun.* **116**, 342.

Pross, H. F. & Baines, M. G. (1976) Spontaneous Human Lymphocyte-mediated Cytotoxicity against Tumour Target Cells. I. The Effect of Malignant Disease. *Int. J. Cancer*, **18**, 593.

Pross, H. F. & Jondal, M. (1975) Cytotoxic Lymphocytes from Normal Donors. A Functional Marker of Human Non-T Lymphocytes. *Clin. exp. Immunol.*, **21**, 226.

Rhim, J. S., Cho, H. Y. & Huebner, R. (1975) Non-producer Human Cells Induced by Murine Sarcoma Virus. *Int. J. Cancer*, **15**, 23.